# Characterizing the nasopharyngeal microbiome and resistome of dairy cattle with and without bovine respiratory disease

**DOI:** 10.1128/spectrum.02648-25

**Published:** 2026-03-24

**Authors:** Adriana Garzon, Craig Miramontes, Bart C. Weimer, Rodrigo Profeta, Alejandro Hoyos-Jaramillo, Heather M. Fritz, Richard V. Pereira

**Affiliations:** 1 Department of Population Health and Reproduction, School of Veterinary Medicine, University of California 70733, Davis, California, USA; 2 100K Pathogen Genome Project, School of Veterinary Medicine, University of California 8789, Davis, California, USA; 3 California Animal Health and Food Safety Lab, University of California 8789, Davis, California, USA; Agriculture and Agri-Food Canada, Lacombe, Canada

**Keywords:** bovine, BRD, antimicrobial resistance, pathobiome, calves

## Abstract

Bovine respiratory disease (BRD) remains a significant economic challenge in dairy cattle despite extensive vaccination programs that have been developed and implemented during the last few decades. This study investigated the nasopharyngeal microbiome and resistome of dairy cattle across various life stages to understand the roles of microbial communities associated with BRD. A case-control study was conducted on three commercial dairy farms in Northern California, collecting nasopharyngeal swabs from 69 animals, including preweaned calves, weaned heifers, and lactating cows with and without BRD. Shotgun metagenomic sequencing was used to characterize both microbiome and resistome profiles observed at the time of BRD diagnosis. Results revealed that BRD is associated with distinct microbial community patterns, rather than the increased abundance of a specific pathogen. Age was a critical factor influencing microbial diversity, with adult cows showing the highest diversity and weaned heifers with BRD showing the lowest. A total of 1,164 bacterial species were identified, with BRD cases harboring 14 unique species compared to control animals. BRD cases were characterized by the co-occurrence of multiple respiratory pathogens, including *Mannheimia haemolytica*, *Pasteurella multocida*, *Histophilus somni*, and *Mesomycoplasma* species, which showed positive correlation with BRD cases but negative correlations in BRD controls, while BRD control animals showed significantly higher abundance of commensal *Staphylococcus* species. Resistome analysis identified 65 antimicrobial resistance genes, with BRD cases harboring more unique resistance genes than BRD controls. These findings challenge traditional single-pathogen models and demonstrate that BRD is likely the result of complex microbial community interactions and changes in community abundance, providing new potential targets to explore when considering prevention strategies toward promoting microbial communities that prevent or reduce the risk of BRD.

**IMPORTANCE:**

Bovine respiratory disease (BRD) represents one of the most economically challenging conditions in cattle production, with an estimated direct cost that exceeds $165 million annually in the United States alone. Despite decades of vaccination efforts targeting known pathogens, BRD prevalence remains unchanged, indicating an incomplete understanding of disease pathogenesis. This study provides critical insights by shifting focus from individual pathogens to entire microbial communities, revealing that BRD involves complex bacterial interactions, as well as the role of the understudied nasal commensal microbiome in healthy animals. The identification of distinct “pathobiomes” associated with disease and protective commensal communities in healthy animals fundamentally changes approaches to BRD prevention and treatment. The discovery that age significantly influences microbiome stability highlights critical intervention periods. Furthermore, the association between BRD and increased antimicrobial resistance genes raises concerns about current treatment and overall management practices, selecting for drug-resistant communities. This research provides a foundation for developing microbiome-based diagnostic tools and interventions supporting healthy microbial ecosystem development.

## INTRODUCTION

Bovine respiratory disease (BRD) is a multifactorial disease affecting cattle populations worldwide, resulting in significant economic losses for the dairy and beef industries (1, 2). The estimated direct cost of BRD in preweaned beef calves on US beef cow-calf operations between 2011 and 2015 was $165 million (3), and the estimated cost per incident case in dairy heifers ranged between $252 and $282 (4). The development of BRD is influenced by complex interactions between the host, the environment, and the respiratory tract microbiome. While the role of the respiratory microbiome in BRD pathogenesis has not been fully elucidated, increasing evidence suggests that the microbial community plays a critical role in maintaining respiratory health and preventing opportunistic pathogen colonization that could result in BRD (5, 6). The nasopharynx serves as a reservoir for potential BRD-associated pathogens, and the composition and dynamics of this microbiome may provide valuable insights into disease risk (7, 8). Understanding the dynamics of the nasopharyngeal microbiota and its associated antimicrobial resistance (AMR) profile (the resistome) is crucial for identifying potential biomarkers for early BRD detection and targeted intervention strategies (8–10).

Previous studies have documented the evolution of the nasopharyngeal microbiota in beef cattle within different scenarios, such as weaning, processing, transportation, and after feedlot arrival (5, 6, 11–13). However, there is a lack of comprehensive data characterizing the microbiome and resistome across different age groups in dairy cattle. This study aims to fill this knowledge gap by investigating the nasopharyngeal microbiome and resistome of preweaned dairy calves, weaned heifers, and lactating cows with and without BRD. We hypothesized that the nasopharyngeal microbiome and resistome of dairy cattle would exhibit distinct compositional profiles that vary by both age group (preweaned calves, weaned heifers, and lactating cows) and BRD status. Specifically, dairy cattle with BRD would demonstrate a dysbiotic nasopharyngeal microbiome characterized by reduced microbial diversity, altered bacterial community structure with increased abundance of opportunistic pathogens (such as *Mannheimia haemolytica*, *Pasteurella multocida,* and *Histophilus somni*), and decreased beneficial commensal bacteria compared to healthy controls within each age group.

## MATERIALS AND METHODS

### Study design

A case-control study was conducted on a convenience sample of three large (>1,000 lactating cows) commercial dairy farms in Northern California. This study was part of a study evaluating BRD bacterial pathogens, where individual preweaned calves (up to 7 weeks of age), weaned heifers, and lactating cows with BRD were matched with control BRD-negative animals (14).

### Disease diagnosis

Monthly, a veterinarian examined all preweaned calves, weaned heifers, and lactating cows from the hospital pen (antimicrobial-treated cows with milk-withhold periods) in participating dairy herds. Clinical evaluations were performed by three trained veterinarians across the participating farms. To minimize inter-observer variability, standardized training sessions with all three veterinarians were conducted before study initiation. BRD-suspect animals underwent age-specific standardized procedures, including clinical assessment, BRD scoring, thoracic auscultation, and lung ultrasound (preweaned calves only). BRD case confirmation followed age-specific criteria. Preweaned calves required a California BRD cumulative score ≥5 (15) and abnormal thoracic auscultation or ultrasound findings (16). Weaned heifers needed a post-weaned dairy calves scoring system cumulative score ≥1 (17), plus abnormal thoracic auscultation. Cows were confirmed with either two primary diagnostic criteria (cough, nasal discharge, increased respiratory effort/rate, fever) or one primary plus one secondary criterion (depressed demeanor, low body condition score). The study enrolled female animals only once and excluded severely depressed, apathetic, unable-to-stand animals, or those lacking permanent identification. For each confirmed case, a BRD-negative control from the same herd, matched by age and breed, was randomly selected. Controls demonstrated normal findings on clinical exams and thoracic auscultation, with preweaned calves also requiring normal lung ultrasound results. Comprehensive records of antimicrobial treatments were not consistently available across participating farms, which represents a limitation of this study.

### Sample collection

Samples were collected from both cases and controls in every age group. Samples were collected using a deep nasopharyngeal swab approach, as previously described (18, 19). Briefly, samples were collected using a single-guarded sterile cotton swab (KI-3000, Kalayjian Industries, Inc., Signal Hill CA, USA) by restraining animals in a standing position, with the animals' nostrils wiped clean with a single-use paper towel and subsequently disinfected with a clean 70% alcohol gauze before inserting the sterile swab medioventrally in the nasal cavity until nasopharyngeal tissue was reached and rotated several times against the mucosa. Swabs were immediately placed in RNase/DNase-free, sterile tubes for DNA extraction. Samples were transported in a cooler with ice to the laboratory for processing.

### DNA extraction and library preparation

Total DNA was extracted from nasopharyngeal swabs within 4 h of sample collection. Cotton tips were removed from the applicators and then placed in 400 μL nuclease-free water and mixed horizontally by vortex for 5 min. The swabs were removed from the microcentrifuge tubes, and the remaining liquid was centrifuged for 5 min at 13,000 × *g* at room temperature (20). The supernatant was discarded, and the DNA was extracted from the pellet using DNeasy PowerSoil Pro Kit (QIAGEN N.V., Hilden, Germany), following the manufacturer’s instructions, with slight modifications. Briefly, for both wash steps, volume washes were increased to 600 µL, left on the membrane for 2 min, and centrifuged for 3 min. DNA concentration was measured at 260 nm using a NanoDrop One^c^ Microvolume UV-VIS spectrophotometer (Thermo Fisher Scientific, Inc., Waltham, Massachusetts, USA). Additionally, for samples with a concentration <50 ng/µL or A260/230 <2.0, DNA was purified using a Genomic DNA Clean & Concentrator-25 kit (Zymo Research Corp., Santa Clara, California, USA).

Sequencing library preparation was conducted in the laboratory of Dr. Bart Weimer (UC Davis) as previously described (21–24). DNA was analyzed on the Agilent 2200 TapeStation System using the Genomic DNA ScreenTape assay for the integrity of gDNA. Libraries were constructed using the KAPA HyperPlus Library Preparation Kit (Roche, Indianapolis, Indiana, USA). Shotgun sequencing was performed using the Illumina NovaSeq 6000 platform with an S4 flow cell (2 × 150 bp) (Illumina Inc., San Diego, California, USA). All raw genome sequences generated in this study are available in the NCBI SRA under the 100K Pathogen Genome Project BioProject (accession number PRJNA1301959).

### Bioinformatics

As previously described (21–24), raw reads were initially trimmed with Trimmomatic (v0.39; command: trimmomatic PE [input] [output] ILLUMINACLIP:[adapters]:2:40:15 LEADING:2 TRAILING:2 SLIDINGWINDOW:4:15 MINLEN:50) (25), and reads were assessed for quality at each step using FastQC (version 0.12.1) (26). Summary reports were compiled using MultiQC (version 1.21) (27). Reads were split into bovine (host) and non-bovine reads using the --classified-out and --unclassified-out options in Kraken2 (version 2.1.3) (28), with a custom database built from three high-quality Bos taurus genomes. These included the bovine genome reference (USDA ARS-UCD1.2, breed Hereford; RefSeq assembly accession: GCF_002263795.3) and two additional representatives from other breeds: GCA_021234555.1 (Jersey) and GCA_021347905.1 (Holstein-Friesian). Microbial reads were identified from non-bovine reads using the classification function in Kraken2 (version 2.1.3) based on the standard Kraken database of microbes from RefSeq, built on 6 July 2023. The “--minimum-hit-groups 3” flag was applied to enhance classification depth and specificity. Microbial reads were calculated to species-level taxa abundances using Bracken (version 2.8) (29). To ensure taxonomic accuracy, prior to downstream species-level analysis, low-abundance taxa were filtered out to ensure a minimum of 100 assigned reads and be present in at least two-thirds of samples.

As previously described (22), antimicrobial resistance genes were analyzed in every genome using ARIBA (version 2.13.3). Antimicrobial resistance genes were screened against the Comprehensive Antibiotic Resistance Database (CARD), the Antibiotic Resistance Gene-Annotation, MEGARes, ResFinder, and the NCBI Antimicrobial Resistance Gene Finder Plus (AMRFinderPlus). The AMR genes were classified according to resistance mechanisms and drug class according to the CARD database and manual curation. AMR determinants were retained for analysis if they fit the minimum criteria of 90% identity and coverage.

### Statistical analysis

Data analysis was conducted using RStudio (version 4.1.2). Independent variables throughout data analysis were BRD status (case or control), age (preweaned calf, weaned heifer, or lactating cow), and the interaction between BRD status and age.

Alpha diversity metrics (Shannon and Simpson indices) were calculated at the species and AMR gene level on a filtered phyloseq object (version 1.48.0), and multiple comparisons were calculated using the Wilcoxon rank-sum test. Relative abundances of bacterial species and AMR genes were calculated in the filtered phyloseq object, and the top 15 more abundant bacteria and genes were plotted using the MicrobiotaProcess (version 1.16.1) package (30).

Center Log-ratio (CLR) was used to normalize reads via the microbiome package (version 1.26.0) (31). For beta-diversity, partial least squares discriminant analysis (PLS-DA) with CLR-normalized distances was used at the species level utilizing MetaboAnalyst (v.6.0) (32). Permutational multivariate analysis of variance (PERMANOVA) based on Euclidean distances with 999 iterations was used to test differences between independent variables. Differential abundance analysis of species between age and BRD status groups was performed using analysis of compositions of microbiomes with bias correction (ANCOM-BC), with p_adj_method set to Benjamini-Hochberg (33). Unique and shared bacterial species and AMR genes were visualized between age and BRD status groups using the UpSetR package (version 1.4.0) (34). Alluvial plots were used to visualize species and AMR genes for >1% abundance at the species and gene level (35).

Bacterial co-occurrence patterns were analyzed using correlation network analysis of CLR-normalized bacterial abundance. Pairwise correlations between all bacterial species were calculated using Spearman’s rank correlation coefficients. Statistical significance of correlations was assessed using a correlation threshold of |r| ≥0.85 and adjusted *P* value <0.05 (Benjamini-Hochberg correction). The resulting correlation matrix was converted into a network format, where nodes represented bacterial species and edges represented significant correlations. The network was visualized and analyzed using Cytoscape (version 3.10.3) software. Network properties, including degree distribution, clustering coefficient, and network density, were calculated to characterize the overall network structure. Community detection was performed using the GLay community clustering algorithm within Cytoscape to identify modules of highly interconnected bacterial species. Node properties were visualized by mapping node size to degree centrality (number of connections) and node color to mean abundance across samples. Edge properties were represented by color (blue for positive correlations, red for negative correlations) and edge width corresponding to correlation strength.

## RESULTS

**Fig 1:**
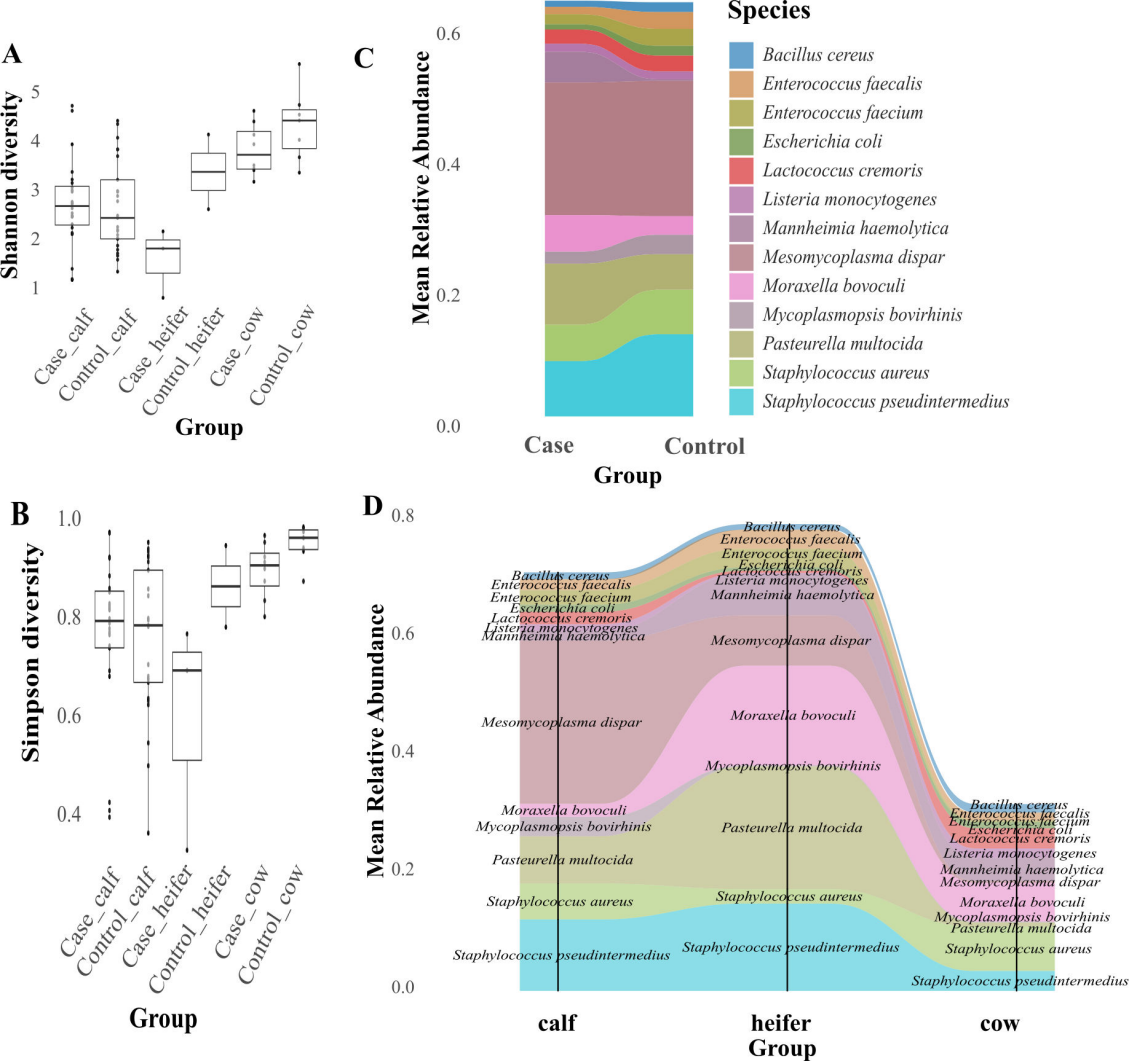
Nasopharyngeal microbiome composition of samples collected from the preweaned calves, weaned heifers, and adult cows from animals with (BRD cases) and without BRD (BRD controls). (**A and B**) Alpha diversity at the species level for (A) Shannon and (B) Simpson diversity indices, comparing BRD cases and BRD healthy controls from three age groups. *P* values represent pairwise comparisons of groups based on the Wilcoxon rank-sum test. *P* < 0.05 was considered a significant difference. (**C and D**) Relative abundance of the top 15 bacterial species by (C) BRD status and (D) age group.

A total of 69 animals were enrolled in this study. The age distribution was similar between BRD cases and controls. Among the 35 BRD cases, 24 animals were preweaned calves (68.5%), 8 animals were cows (22.8%), and 3 animals were weaned heifers (8.7%). The 34 control animals showed a comparable distribution, with 25 calves (73.5%), 7 cows (20.6%), and 2 heifers (5.9%). Microbial alpha diversity indices at the species level did not significantly differ between BRD status (Fig. S1A and B). However, Shannon and Simpson diversity indices statistically differed between age groups (*P* < 0.001), with higher alpha diversity observed in both control cows and heifers, and the lowest in heifer cases (Fig. 1A and B). Mean relative abundance plots demonstrated a high diversity of bacterial composition, which varied between BRD status (Fig. 1C) and by age group (Fig. 1D). The mean relative abundance of *Mannheimia haemolytica, Pasteurella multocida, and Moraxella bovoculis* was higher in BRD cases, while *Staphylococcus pseudintermedius* and *Mycoplasmopsis bovorhinis* mean abundance was higher in BRD control animals. Overall, total bacterial relative abundance was higher in weaned heifers and calves and decreased in adult cows.

**Fig 2:**
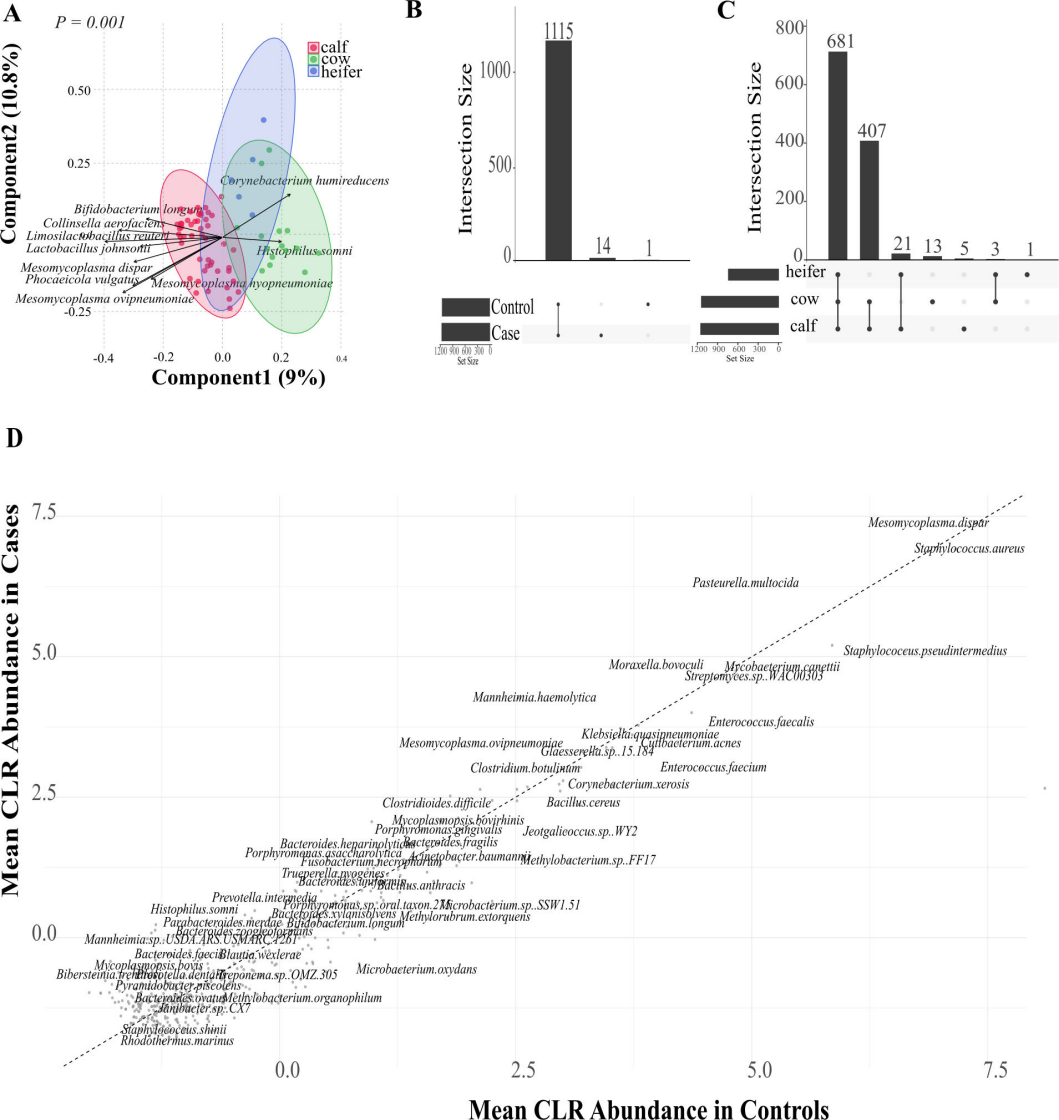
Nasopharyngeal microbiome analysis of samples collected from the preweaned calves, weaned heifers, and adult cows from animals with (BRD cases) and without BRD (BRD controls). (**A**) PLS-DA of the center log ratio normalized bacterial species for age groups. *P* value represents PERMANOVA test with *P* < 0.05 considered a significant difference. (**B and C**) Upset plots for the intersection of shared and unique bacterial species between (B) BRD status and (C) age group. (**D**) Pairwise comparison scatterplot for the center log ratio normalized mean relative abundance at the species level between BRD cases and BRD controls.

Composition analysis revealed substantial diversity and distinct patterns of organism abundance across age groups and BRD status. For beta diversity, two ordinations were created to evaluate differences between BRD status (Fig. S1C) and age groups (Fig. 2A), identifying *Mesomycoplasma hyopneumoniae, Mesomycoplasma ovipneumoniae, Mesomycoplasma dispar,* and *Histophilus somni* as important predictor variables when evaluating differences between age groups (Fig. S1D), while *H. somni*, *Mesomycoplasma ovipneumoniae,* and *Moraxella ovis* were important predictor variables when evaluating differences between BRD groups (Fig. S1E). PERMANOVA analysis revealed no significant differences in microbial diversity between BRD status (*P* = 0.23); however, there was a significant difference between age groups (*P* = 0.001). Similarly, we identified 1,130 bacterial species across samples. BRD cases and controls shared 1,115 unique species, while 14 unique species were identified in BRD cases, and samples from BRD controls had only one exclusive species (Fig. 2B). When unique species were compared among age groups, 681 species were shared among adult cows, weaned heifers, and preweaned calves. Adult cows showed the highest number of unique species (*n* = 13), while preweaned calves (*n* = 5) and weaned heifers (*n* = 1) had a lower number of unique species. Also, while adult cows and preweaned calves shared 408 unique species, adult cows and weaned heifers only shared 3 unique species (Fig. 2C).

ANCOM-BC was used to investigate variations in microbiome composition across BRD status and age groups. This analysis revealed that *Mesomycoplasma dispar* was the only differentially abundant bacterial species, with high abundance in cases and control preweaned calves and in cases from weaned heifers, while the abundance was low in cases and control adult cows, as well as control weaned heifers (Fig. S1F). A comparison of the mean relative abundance of bacteria among BRD status (Fig. 2D) was conducted to evaluate differences in the abundance of multiple bacteria at a community level. The scatterplot analysis revealed that while most bacterial species maintained similar relative abundances regardless of disease status, several species clustered above the diagonal line in the upper right quadrant, indicating higher abundance in BRD cases compared to control animals. Notable bacteria with elevated abundance in BRD cases included *P. multocida*, *M. dispar*, *M. ovipneumoniae*, and *M. haemolytica*.

**Fig 3:**
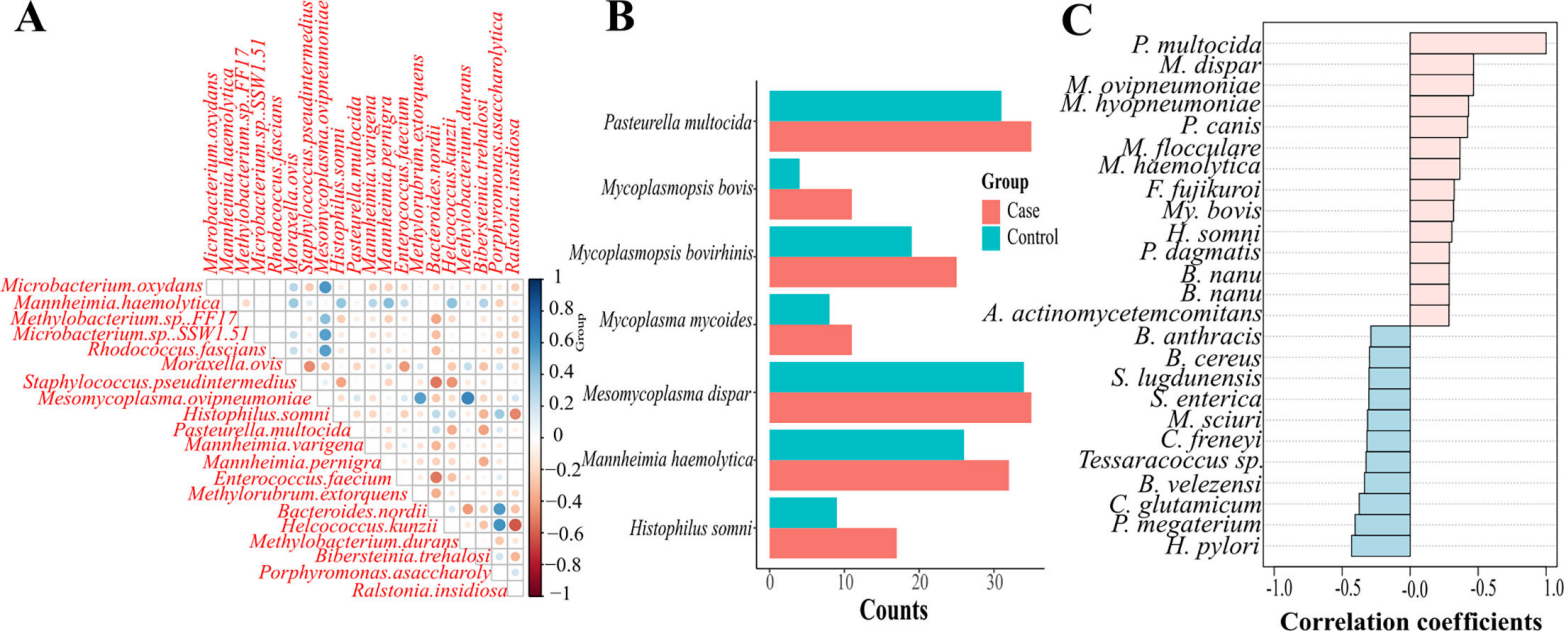
Nasopharyngeal microbiome analysis of samples collected from the preweaned calves, weaned heifers, and adult cows from animals with (BRD cases) and without BRD (BRD controls). (**A**) Correlation plot of the difference in bacterial species correlation coefficients between BRD cases and BRD controls. (**B**) Prevalence of seven bacterial species associated with BRD according to BRD status. (**C**) Top 25 bacterial species correlated with *P. multocida* in BRD cases compared to BRD controls.

Network analysis of bacterial species revealed multiple correlations of bacteria between BRD status (Fig. S2), highly driven by bacterial genus. Two distinct clusters of bacterial co-occurrences were observed. A co-occurrence group of *Mesomycoplasma* species (*M. dispar, M. flocculare,* and *M*. *hyopneumoniae*) was observed in both BRD cases and controls, with opposite correlation directions (positively correlated in BRD cases and negatively correlated in BRD controls). A second group of bacterial co-occurrences was observed among three *Mannheimia* species (*M. varigena, M. bovis,* and *Mannheimia* spp. USDA-ARS-USMARC-1261). Pairwise co-occurrence analysis of bacterial species revealed 20 statistically significant correlations in both BRD cases and controls (Fig. S3A and B). When the co-occurrence was compared between BRD status, multiple pairwise comparisons were statistically positively correlated in the BRD groups, predominantly with *M. ovipneumoniae,* while recognized environmental bacteria were mostly correlated with BRD controls, such as *Ralstonia insidiosa* (Fig. 3A).

To evaluate the prevalence of the main bacteria associated with BRD, we evaluated the presence/absence of these bacteria in relation to the BRD status groups. This analysis revealed that the prevalence of *P. multocida, M. bovis, M. bovirhinis, M. mycoides*, *M. haemolytica,* and *H. somni* was higher in BRD cases compared to controls (Fig. 3B). Based on the high prevalence of *P. multocida* regardless of BRD status, we used *P. multocida* as a bacterial indicator to evaluate if the co-occurrence of other species with *P. multocida* differed by BRD status. These results evidenced that in BRD cases, multiple bacteria that were positively correlated with *P. multocida* are recognized bacteria associated with BRD development, including *M. haemolytica, H. somni,* and multiple *Mesomycoplasma* species (Fig. 3C), suggesting that specific bacterial community members co-occur based on health status.

**Fig 4:**
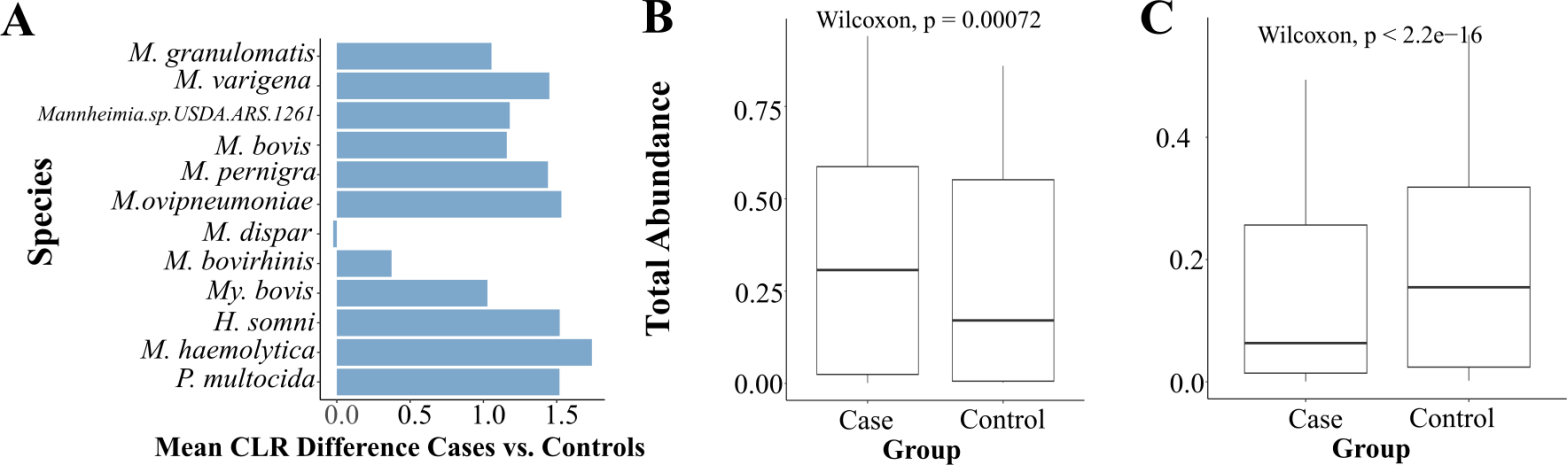
Nasopharyngeal microbiome analysis of samples collected from the preweaned calves, weaned heifers, and adult cows from animals with (BRD cases) and without BRD (BRD controls). (**A**) Difference in the center log ratio mean abundance of BRD cases compared to BRD controls. (**B and C**) Total abundance for (B) BRD-associated bacterial species and (C) commensal *Staphylococcus* bacteria associated with BRD status.

CLR-transformed bacterial abundance data revealed significant compositional differences between BRD cases and BRD controls when comparing bacteria relevant for BRD development (Fig. 4A). BRD cases showed marked enrichment of key respiratory pathogens, with *M. haemolytica* exhibiting the greatest increase in relative abundance (CLR mean abundance difference: 1.7), followed by *P. multocida* (CLR mean abundance difference: 1.5), *H. somni* (CLR mean abundance difference: 1.5), and *M. ovipneumoniae* (CLR mean abundance difference: 1.5). Additional *Mannheimia* species, including *M. granulomatis*, *M. varigena,* and *M. bovis*, demonstrated moderate but consistent increases in BRD cases (mean CLR differences: 0.3–0.5). Conversely, BRD cases exhibited reduced abundance of *Mesomycoplasma bovirhinis, M. dispar,* and *Moraxella* species compared to controls.

To further test the hypothesis that the relative abundance of specific microbial community members changes in abundance with health status, we compared the overall mean relative abundance of bacteria positively correlated with BRD as a collective community rather than as individual bacterial species. The results showed that BRD cases had a significantly higher abundance of this microbial community compared to controls (*P* < 0.001) (Fig. 4B). To validate our community-based hypothesis, we contrasted the abundance of this disease-associated community with that of *Staphylococcus*, a bacterial genus widely recognized as a normal commensal inhabitant of the cattle nasopharynx. Within this genus, we identified 52 different *Staphylococcus* species, such as *S. pseudintermedius*, *S. aureus, S. agnetis, S. argenteus, S. arlettae, and S. auricularis*, among others. This analysis revealed the opposite pattern: the commensal *Staphylococcus* community was significantly more abundant in BRD controls compared to BRD cases (*P* < 0.0001) (Fig. 4C).

**Fig 5:**
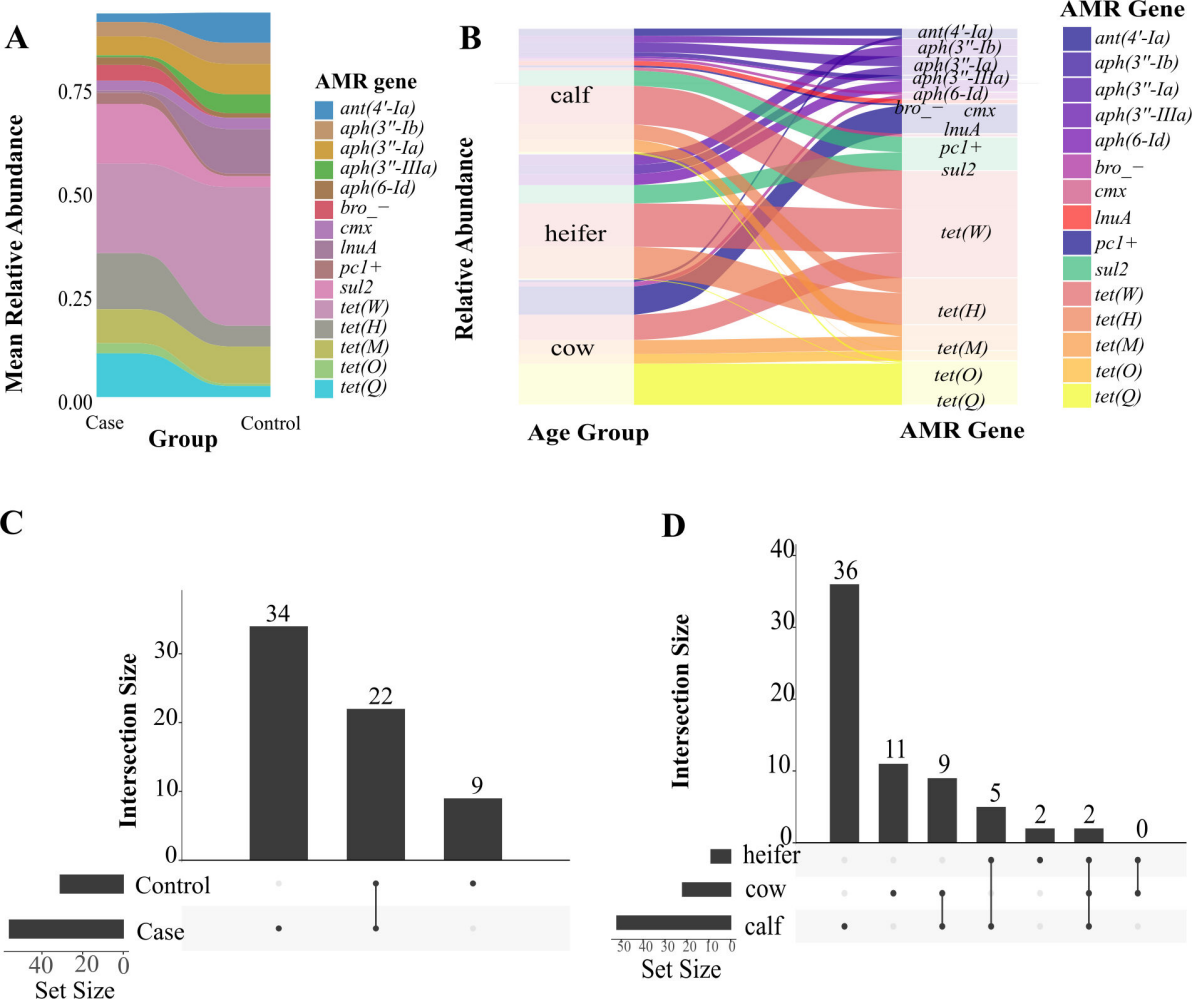
Antimicrobial resistance genes in the preweaned calves, weaned heifers, and adult cows from animals with (BRD cases) and without BRD (BRD controls). (**A and B**) Relative abundance of the top 15 antimicrobial resistance genes by (A) BRD status and (B) age group. (**C and D**) Upset plots for the intersection of shared and unique antimicrobial resistance genes between (C) BRD status and (D) age group.

The resistome analysis revealed distinct patterns in gene abundance across BRD status (Fig. 5A) and age groups (Fig. 5B). We identified 65 AMR genes that confer resistance to 18 different antimicrobial drug classes, with tetracycline AMR genes being the most prevalent type of genes across age and BRD status. Upset plots showed the number of unique and shared genes between BRD status and age groups (Fig. 5C and D). Particularly, 22 genes were shared among BRD status, with cases (*n* = 34) having the highest number of unique AMR genes compared to control animals (*n* = 9). Correspondingly, only two genes were shared among the three age groups, with preweaned calves having the highest number of unique genes (*n* = 36), followed by adult cows (*n* = 11), and weaned heifers having the lowest number of unique AMR genes (*n* = 2).

## DISCUSSION

This study used shotgun sequencing to characterize the microbiome and resistome of nasopharyngeal samples from preweaned calves, weaned heifers, and adult dairy cows, examining their association with BRD status and age group. This research represents one of the first comprehensive investigations of nasopharyngeal microbiome-resistome dynamics across different life stages in dairy cattle. Findings from this study reveal the complex associations between the nasopharyngeal microbiome composition, age, and BRD status in dairy cattle. While alpha diversity metrics did not significantly differ by BRD status, age emerged as a critical factor influencing microbial diversity patterns. The higher diversity in adult cows, irrespective of disease status, and the decrease in diversity indices in weaned heifers, with the lowest diversity in BRD cases among heifers, suggests that age-related immune development and environmental exposure play crucial roles in shaping respiratory microbiome stability. This age-related diversity pattern differs from previous studies, in which diversity was higher at birth but decreased as calves aged (36, 37), attributed to a more homogenous and less diverse microbiome establishment. The higher alpha diversity in adult cows may reflect their extended exposure to diverse environmental microorganisms and the maturation of their immune systems, allowing for the establishment of more complex and stable microbial communities. Conversely, the reduced diversity in younger animals, particularly diseased heifers, may indicate a less stable microbiome that is more susceptible to pathogen colonization and disease development. Moreover, the results from this study also differ from previous studies that indicate that alpha diversity is significantly lower in BRD cases compared to healthy animals (38–40), further reinforcing our findings that the nasopharyngeal microbiome is a multi-organismal disease rather than a single pathogen dominating the population to cause BRD.

The differences in unique and shared bacterial species across age groups provide insights into the respiratory microbiome establishment and maturation. Adult cows exhibited the highest number of unique species (*n* = 13), while preweaned calves and weaned heifers had substantially fewer unique species. This pattern further reinforces our findings, suggesting that microbiome complexity might increase with age, potentially contributing to enhanced disease resistance in mature animals. The limited species overlap between adult cows and weaned heifers (three shared species) compared to adult cows and preweaned calves (408 shared species) indicates that the weaning period may represent a critical period for microbiome restructuring. This finding may explain the increased BRD susceptibility often observed during the weaning period and highlights the importance of management strategies that support microbiome stability and maturation during this critical stage. While these findings suggest strong age-related patterns in microbial community structure, the weaned heifer group’s small sample size requires cautious interpretation. Further validation in larger studies is warranted to validate age-specific effects on the nasopharyngeal microbiome in the context of BRD.

The identification of distinct microbial communities associated with BRD status represents a significant finding that challenges the traditional single-pathogen disease model (41–43). Our results demonstrate that BRD cases are characterized by the co-occurrence of multiple respiratory pathogens, including *M. haemolytica*, *P. multocida*, *H. somni*, and various *Mesomycoplasma* species. The strong positive correlations between these pathogens in diseased animals, contrasted with their negative correlations in BRD controls, suggest that BRD development involves the establishment of pathogenic microbial networks rather than simple pathogen overgrowth. The CLR-transformed abundance analysis revealed *M. haemolytica* as the most enriched pathogen in BRD cases, with a mean abundance difference of 1.7, followed by *P. multocida* and *H. somni*. This finding supports the established role of these bacteria as primary BRD pathogens while highlighting their synergistic relationships within the disease-associated microbiome. The consistent co-occurrence of these pathogens with *P. multocida* in diseased animals further supports the concept of a “pathobiome,” defined as a community of microorganisms that collectively contribute to disease pathogenesis (44). Our results are further supported by the finding that healthy animals harbor distinct microbial communities, which may protect against BRD development. The significantly higher abundance of *Staphylococcus* species in BRD healthy controls suggests that these commensal bacteria may play a protective role by competing with pathogens for colonization sites or producing antimicrobial compounds, as has been shown in mouse models and extrapolated to respiratory diseases in humans (45), as well as digestive health (46). The inverse relationship between pathogen-associated communities and commensal *Staphylococcus* communities provides evidence for competitive mechanisms in the dairy cattle nasopharynx. Furthermore, the high abundance of environmental bacteria, such as *R. insidiosa*, which showed stronger correlations in BRD controls, indicates that exposure to diverse environmental microorganisms may contribute to microbiome development and maturation. This finding has important implications for management practices, and further research is warranted to investigate the effect of environmental bacteria on the respiratory microbiome and disease susceptibility. These findings have several important implications for BRD prevention and treatment strategies. The identification of pathogen-associated microbial communities suggests that diagnostic approaches should consider overall microbiome composition and changes in overall abundance as a community, rather than focusing solely on individual pathogens. While our study identifies microbial signatures associated with BRD at the time of clinical diagnosis, longitudinal studies are needed to validate these findings. Prospective sampling of animals from birth through various life stages would enable the determination of whether the microbial patterns observed in this study precede disease onset or result from the disease process. Such studies could also reveal whether specific microbiome trajectories predict individual animals' susceptibility to BRD, enabling targeted prevention strategies for high-risk individuals. Microbiome-based diagnostics could potentially identify animals at risk for BRD development before clinical signs appear. The strong association between commensal bacteria and health status supports the development of management practices that promote beneficial microbiome establishment. However, it is important to note that while correlation-based network analysis reveals patterns of bacterial co-occurrence, these statistical associations do not necessarily indicate direct microbial interactions. Further studies are needed to validate mechanistic relationships.

The resistome analysis provided insight into both age-related differences and disease-associated changes in AMR abundance. The predominance of tetracycline resistance genes across all groups aligns with the widespread use of tetracyclines in cattle production, aligning with previous research in which tetracyclines were the most common antimicrobial resistance class found (47–49). BRD cases harbored a higher number of unique AMR genes (*n* = 34) compared to controls (*n* = 9), suggesting that disease-associated microbiomes may serve as reservoirs for resistance genes. This finding has significant implications for antimicrobial stewardship, as BRD treatment may be one of the factors contributing to selection for multi-resistant bacterial communities. The age-related distribution of AMR genes, with preweaned calves showing the highest number of unique genes (*n* = 36), agrees with previous studies in which antimicrobial resistance abundance decreases with age (50–52), indicating that early antimicrobial exposure may have lasting effects on resistome development, which highlights the impact of BRD in young animals. The lack of a complete antimicrobial treatment history represents a limitation of our study. We cannot definitively rule out the possibility that previous antimicrobial exposures may have influenced the nasopharyngeal microbiome or resistome profiles. Future studies should incorporate systematic antimicrobial treatment history documentation to better account for potential effects of prior exposures on microbiome and resistome profiles.

### Conclusion

This study demonstrates that BRD is associated with distinct patterns in the nasopharyngeal microbial community, rather than with changes in the abundance of specific pathogens, indicating that it is likely a polymicrobial condition. The identification of age-related microbiome development patterns and the protective role of commensal bacteria provides new targets for disease prevention strategies. The integration of microbiome and resistome data highlights the complex interplay between microbial communities, antimicrobial resistance, and disease development, emphasizing the need for holistic approaches to BRD management that consider the entire microbial ecosystem rather than specific pathogens alone.

## Data Availability

All raw genome sequences generated in this study are available at the 100K Pathogen Genome Project BioProject (NCBI PRJNA186441) under BioProject accession number PRJNA1301959.

## SUPPLEMENTAL MATERIAL

Supplemental figures spectrum.02648-25-s0001.docx Fig. S1 to S3.

ASM does not own the copyrights to Supplemental Material that may be linked to, or accessed through, an article. The authors have granted ASM a non-exclusive, world-wide license to publish the Supplemental Material files. Please contact the corresponding author directly for reuse.
