# Supplementary material for: Characterizing the nasopharyngeal microbiome and resistome of dairy cattle with and without bovine respiratory disease
Source: Microbiol Spectr. 2026 Mar 24;14(5):e02648-25. doi: 10.1128/spectrum.02648-25 (PMC13141890; doi:10.1128/spectrum.02648-25)
Supplement: Supplemental figures — Fig. S1 to S3. [file spectrum.02648-25-s0001.docx]

**Characterizing the nasopharyngeal microbiome and resistome of dairy cattle with and without Bovine Respiratory Disease**

Adriana Garzon^1^, Craig Miramontes^1^, Bart C Weimer^1,2*^, Rodrigo Profeta^1,2^, Alejandro Hoyos-Jaramillo^1^, Heather M Fritz^3^, Richard V Pereira^1^*

^1^Department of Population Health and Reproduction, School of Veterinary Medicine, University of California, Davis, CA, USA

^2^100K Pathogen Genome Project, School of Veterinary Medicine, University of California, Davis, CA, USA

^3^California Animal Health and Food Safety Lab, University of California, Davis, CA, USA

*** Correspondence:**Richard Pereira
rvpereira@ucdavis.edu

Bart C Weimer
bcweimer@ucdavis.edu

**Supplemental Figure 1.** Nasopharyngeal microbiome analysis of samples collected from the preweaned calves, weaned heifers and adult cows from animals with (BRD cases) and without BRD (BRD controls). Alpha diversity at the species level for Shannon and **B.** Simpson diversity indexes, comparing BRD cases and BRD controls. *P-*values represent pairwise comparisons of groups based on the Wilcoxon Sum Rank Test. **C.** Partial Least Squares Discriminant Analysis (PLS-DA) of the center log ratio normalized bacterial species for BRD status. P-value represent PERMANOVA test. *P* < 0.05 was considered a significant difference. **D.** Top 15 influential bacterial species for PLS-DA for BRD status, and **E.** Age groups, and **F.** Relative abundance for the AMCOM-BC test identifying *M. dispa*r as a differentially abundant species among BRD cases and BRD control within age groups.


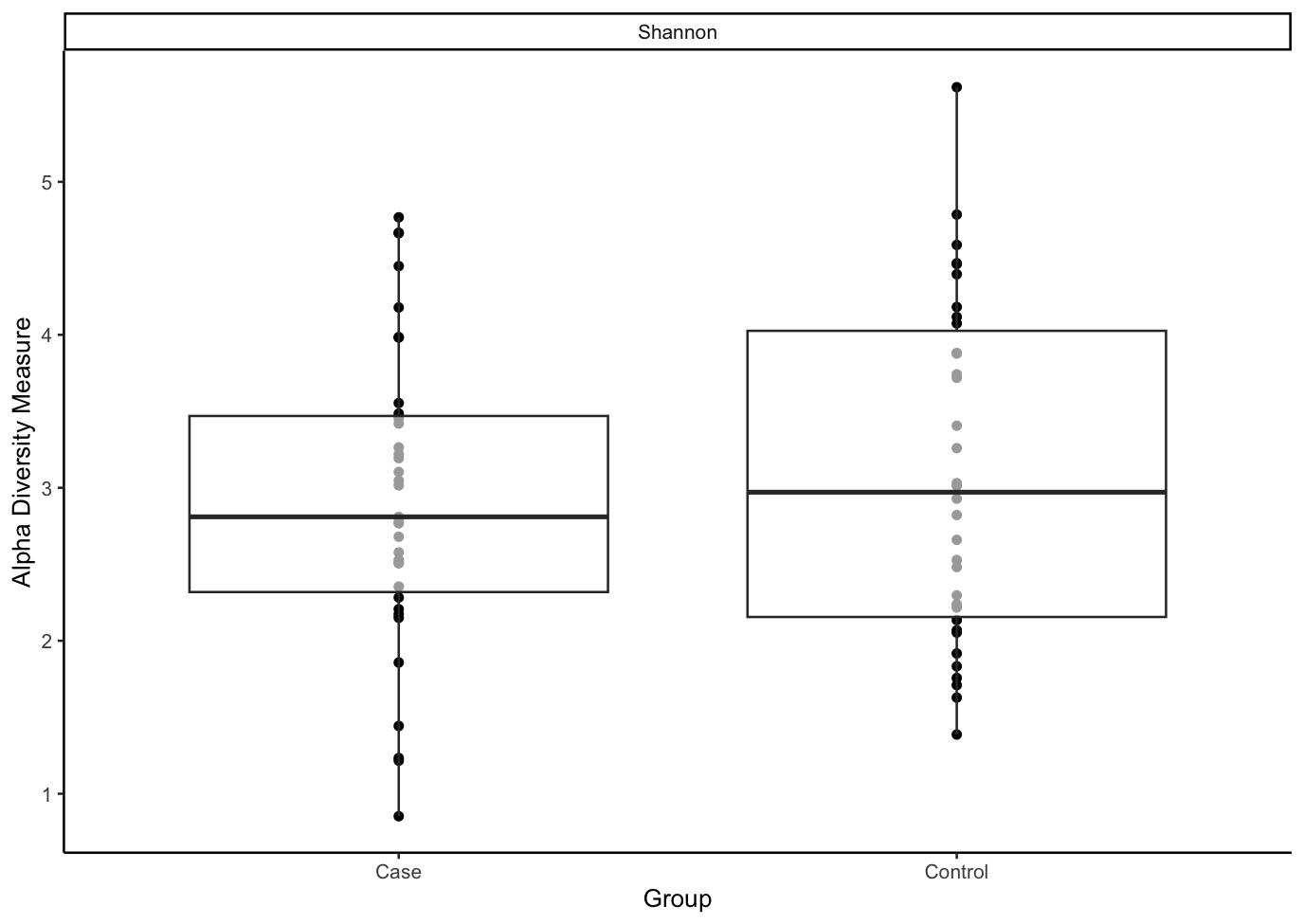

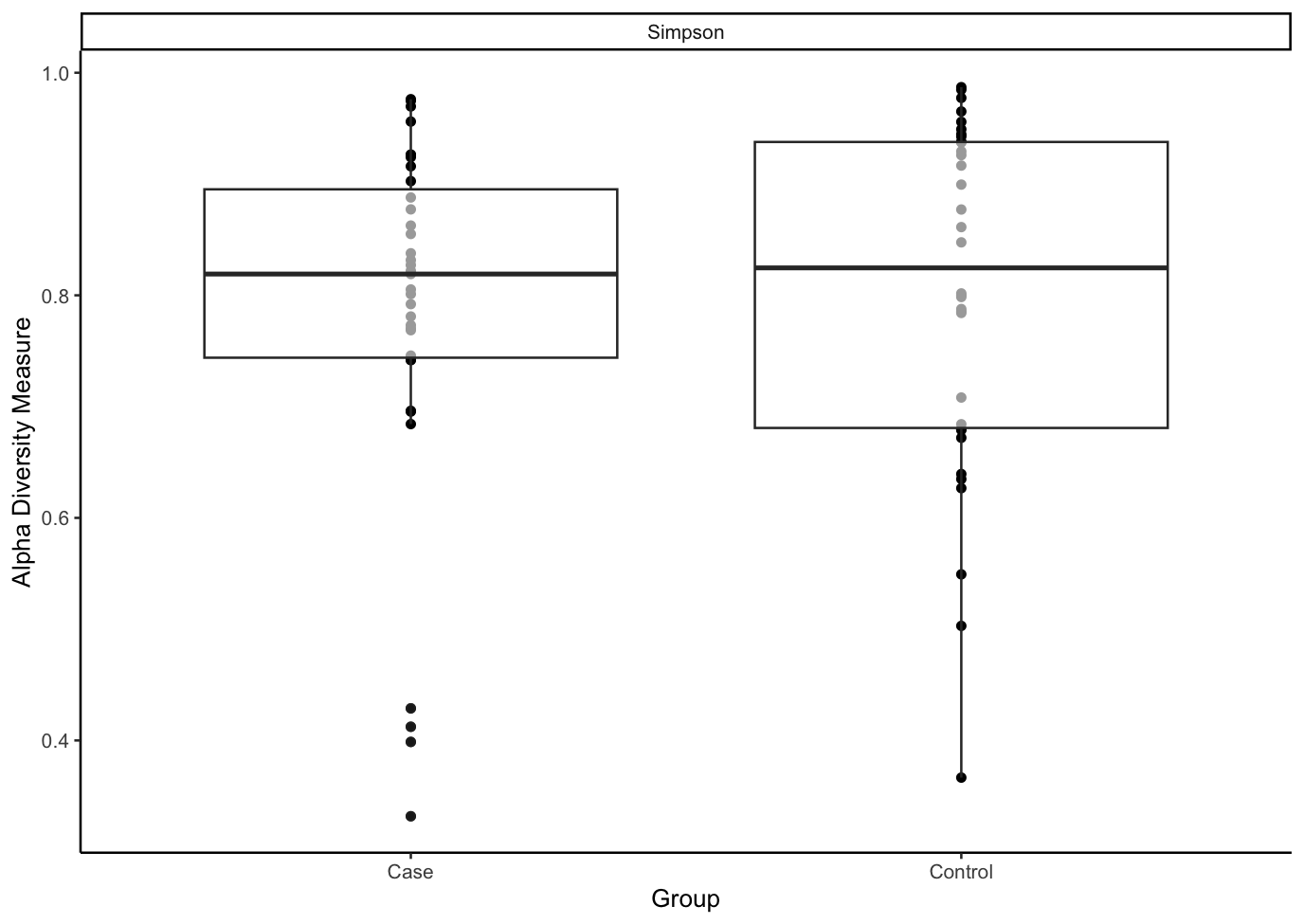


**A**


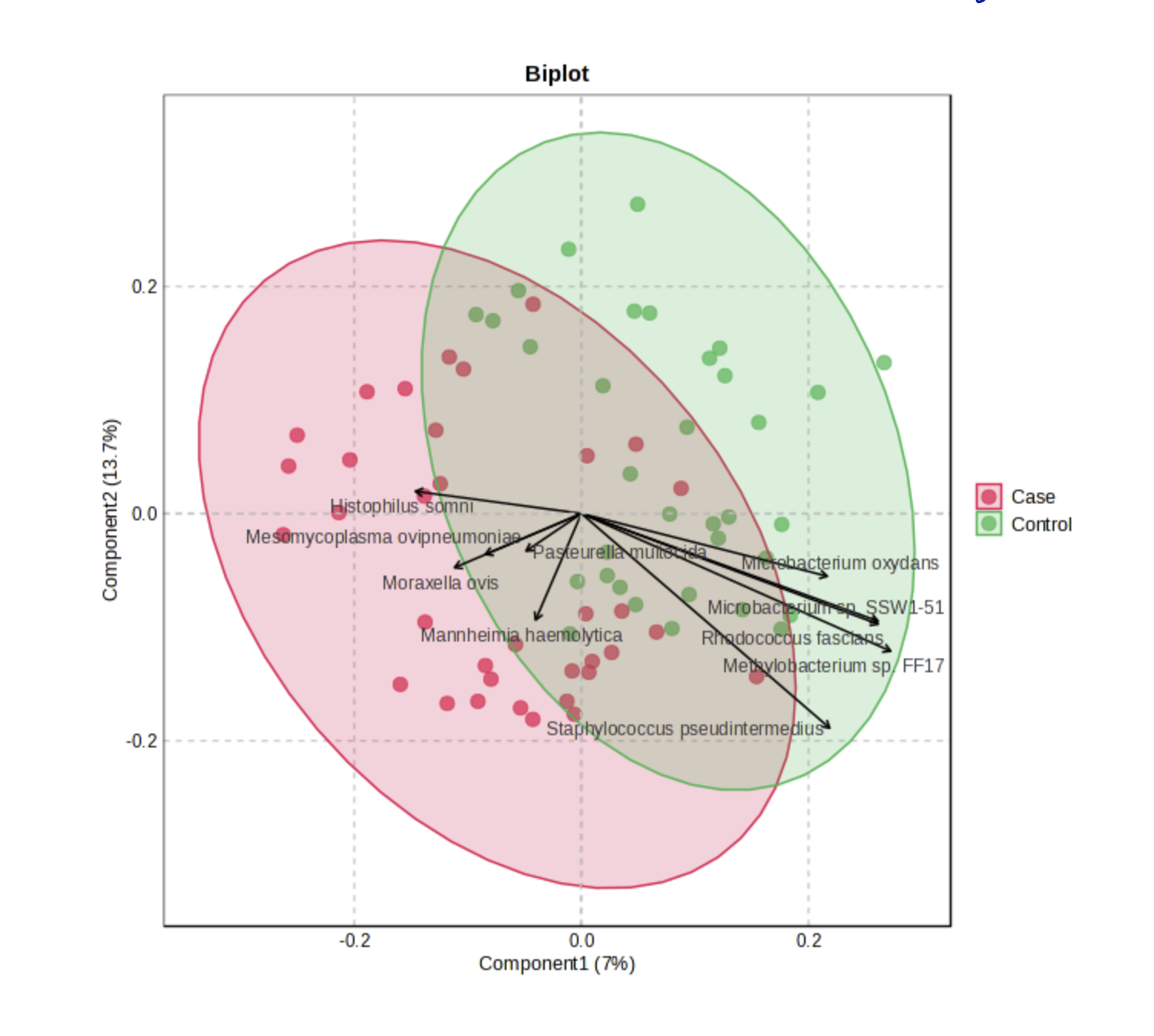


**B**

**C**


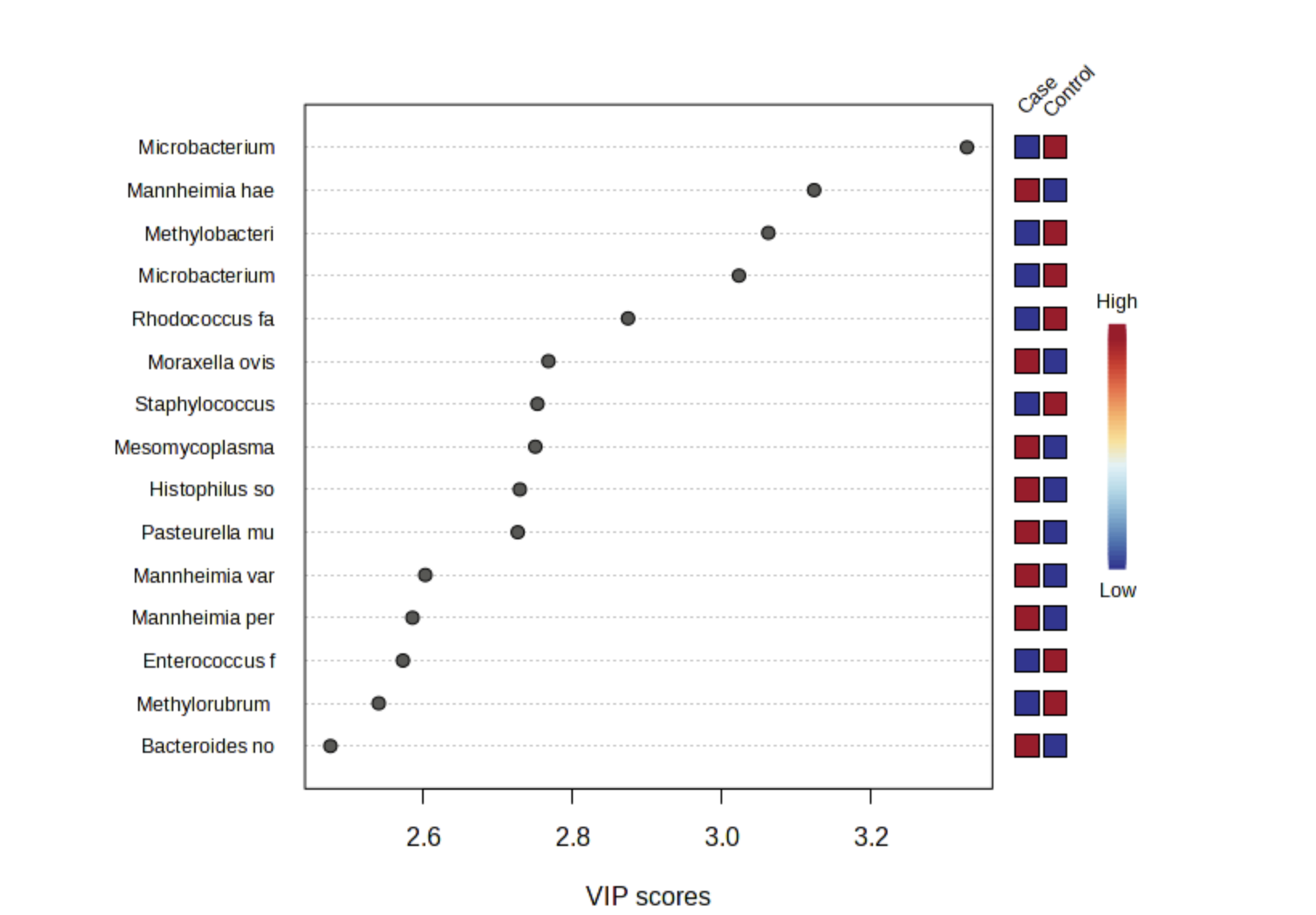

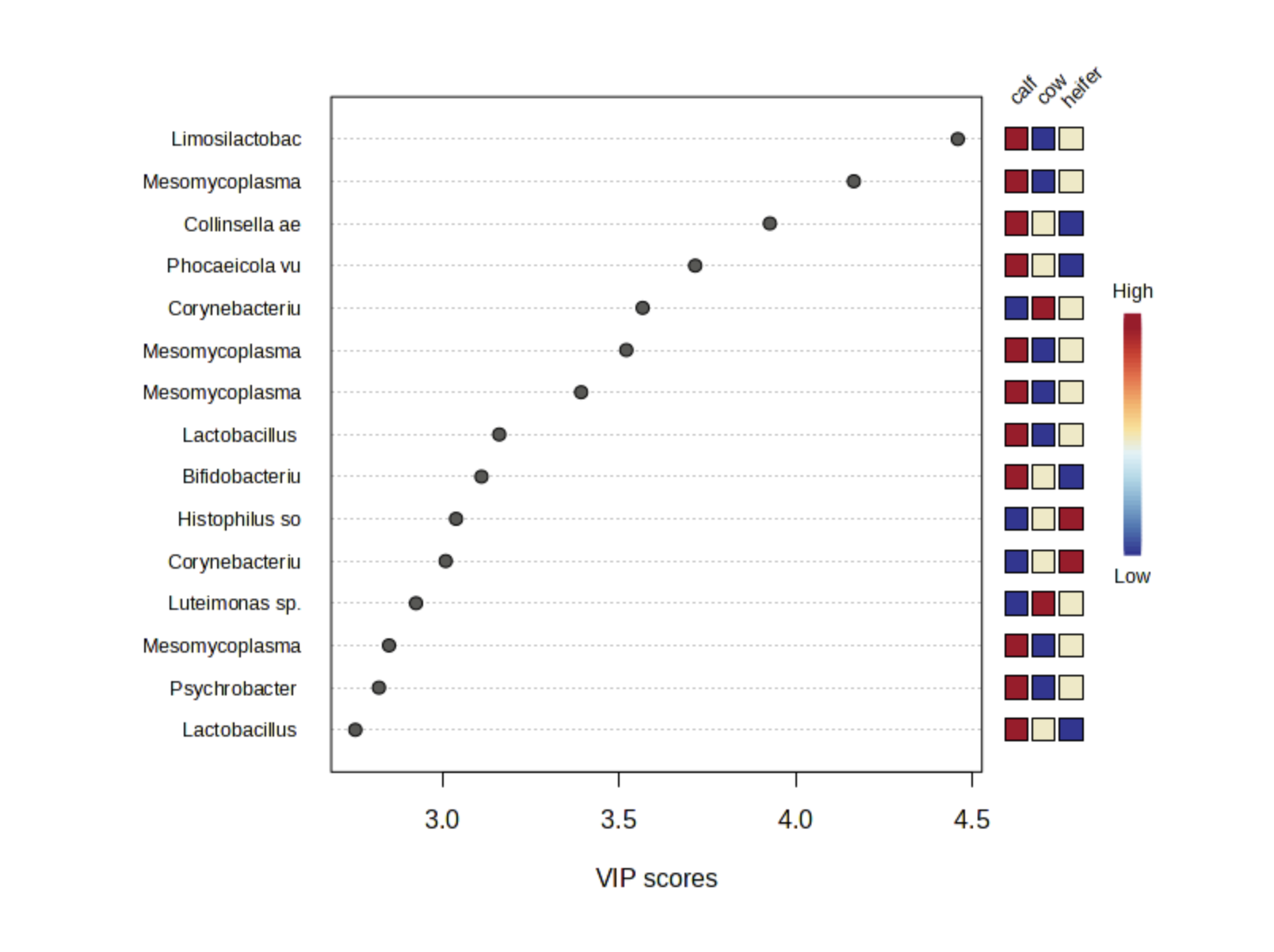

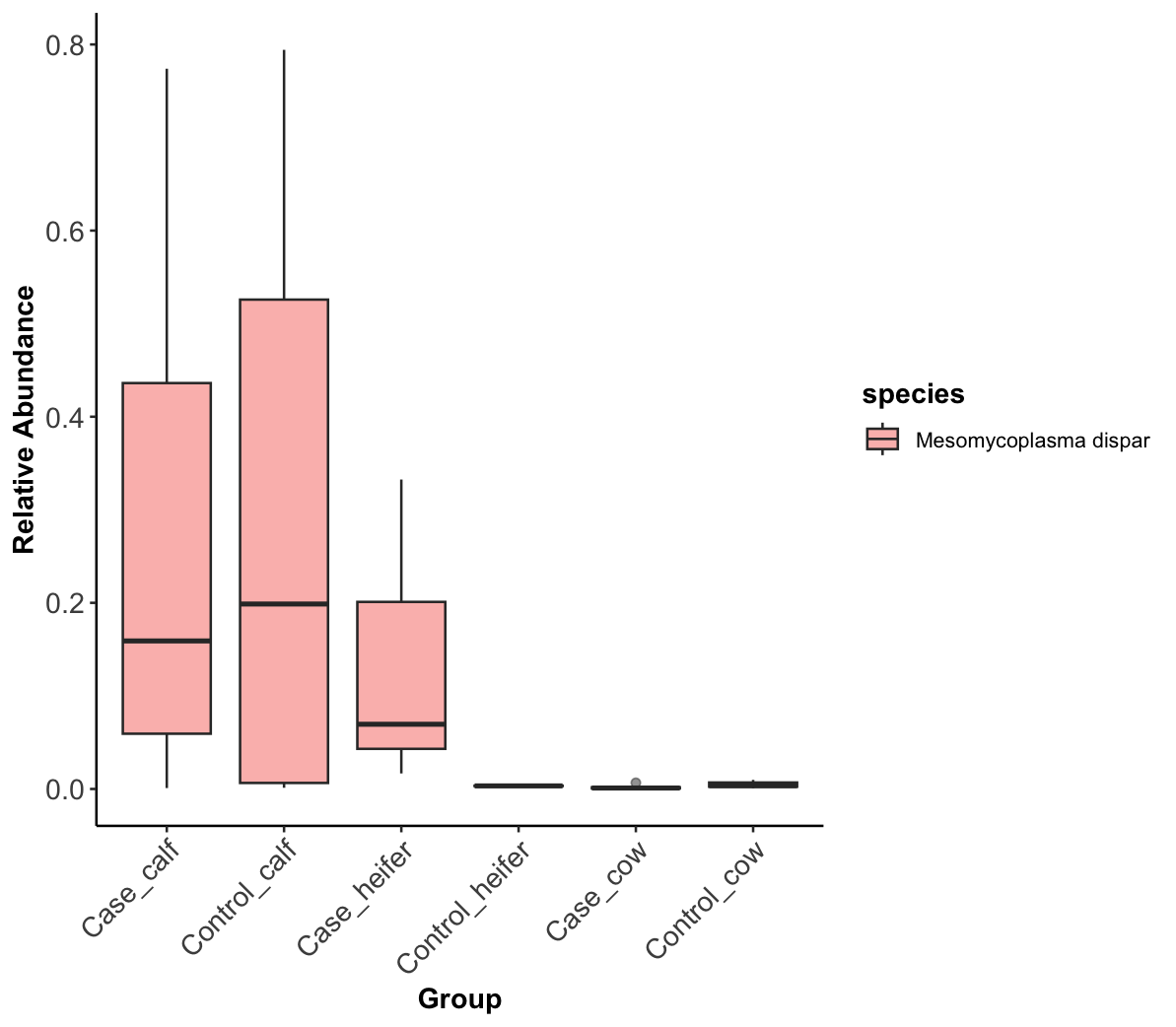


**D**

**E**

**F**

P =0.23


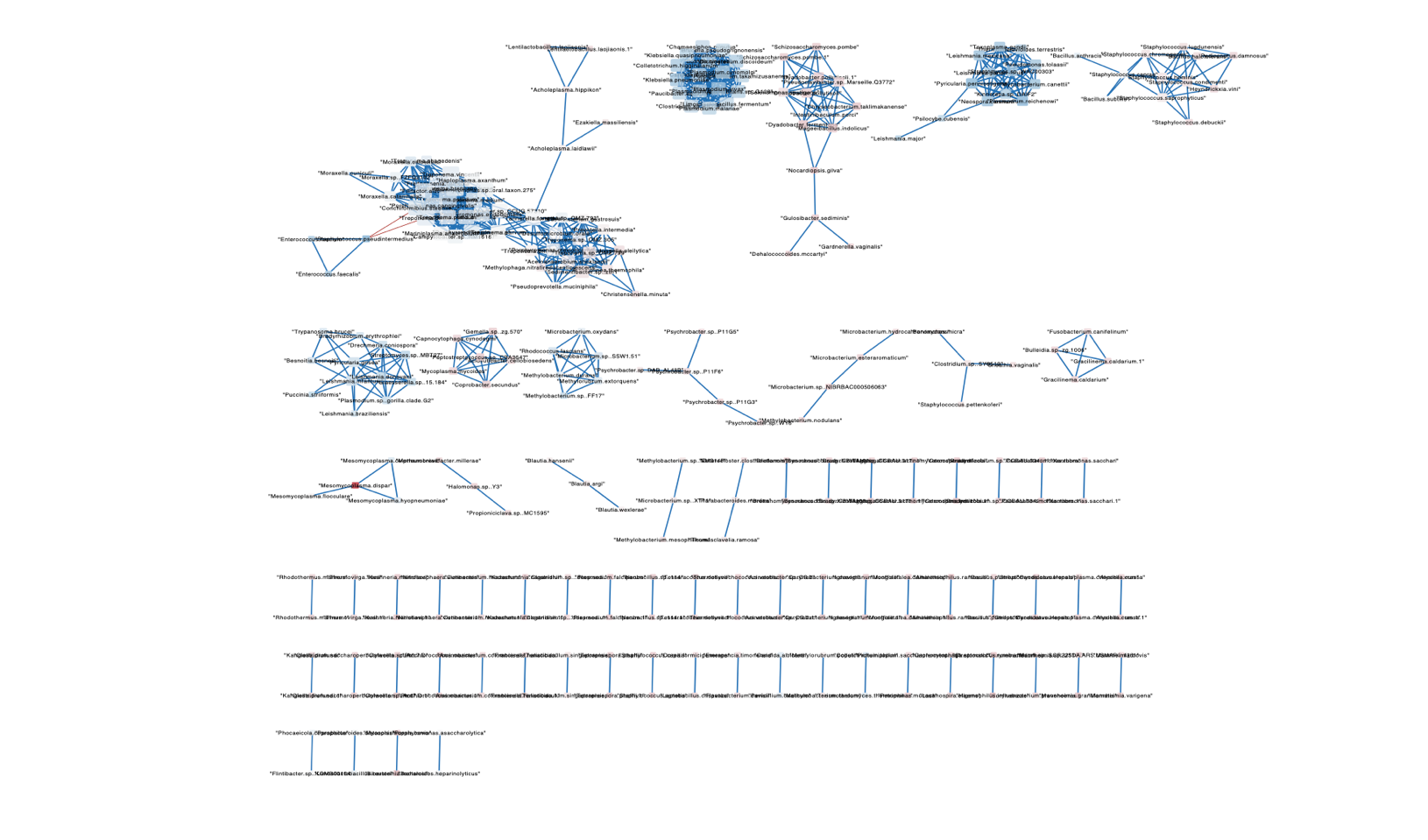
**Supplemental Figure 2.** Nasopharyngeal microbiome analysis of samples collected from the preweaned calves, weaned heifers and adult cows from animals with (BRD cases) and without BRD (BRD controls). **A.** Co-occurrence network of bacterial species in BRD control and **B.** BRD cases.

**A**


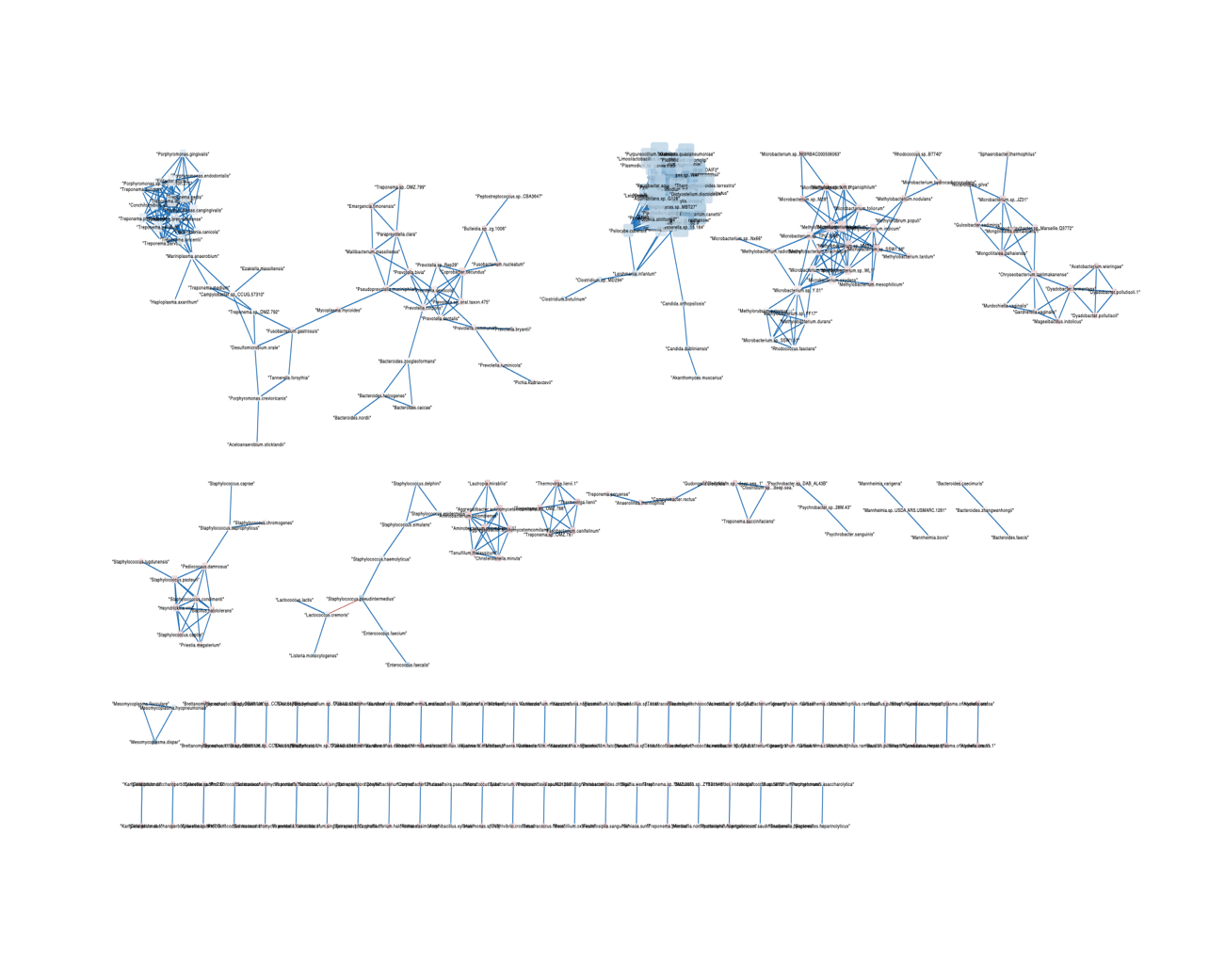


**B**


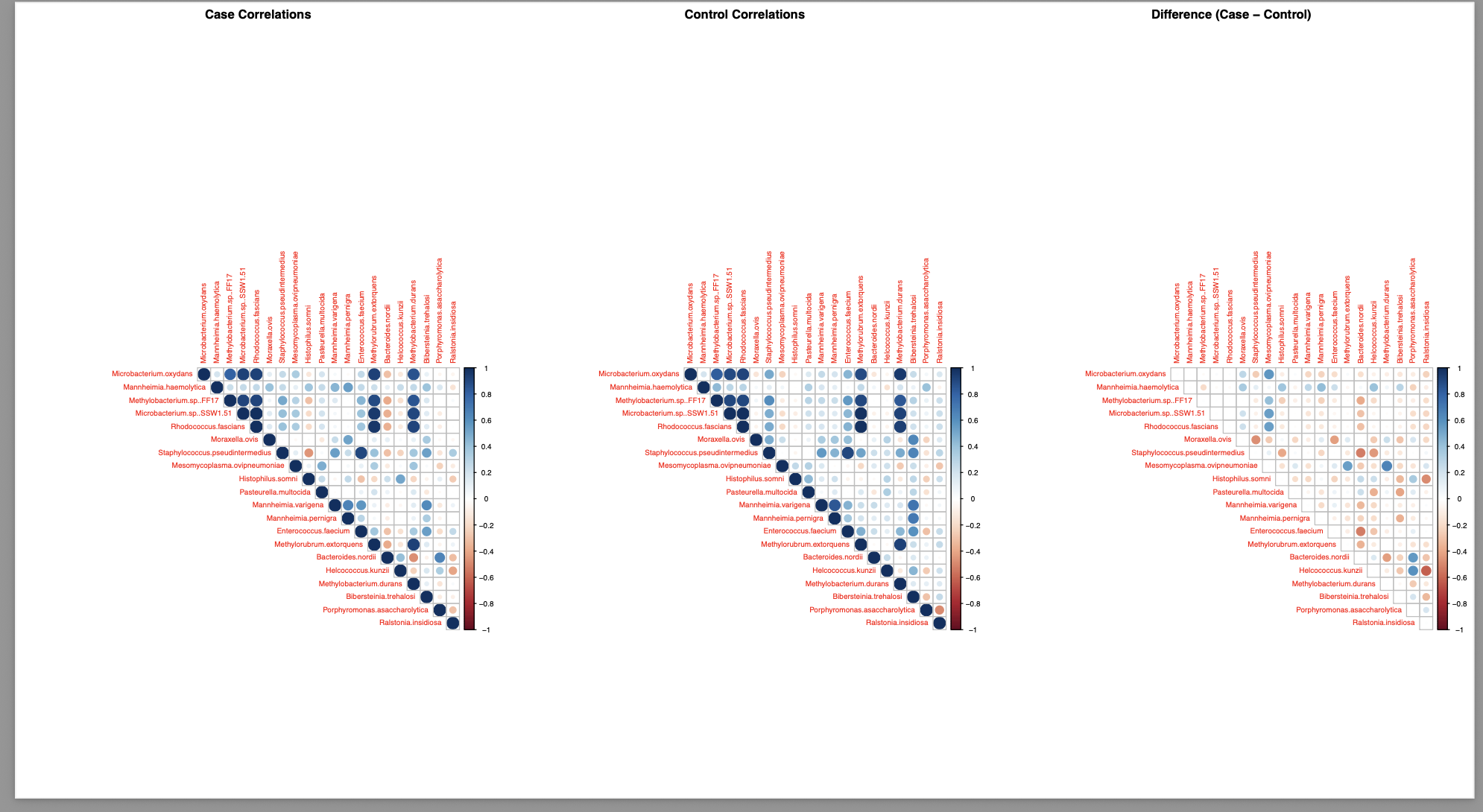
**Supplemental Figure 3.** Nasopharyngeal microbiome analysis of samples collected from the preweaned calves, weaned heifers and adult cows from animals with (BRD cases) and without BRD (BRD controls). **A.** Correlation plot of bacterial species in BRD cases and **B.** BRD controls.


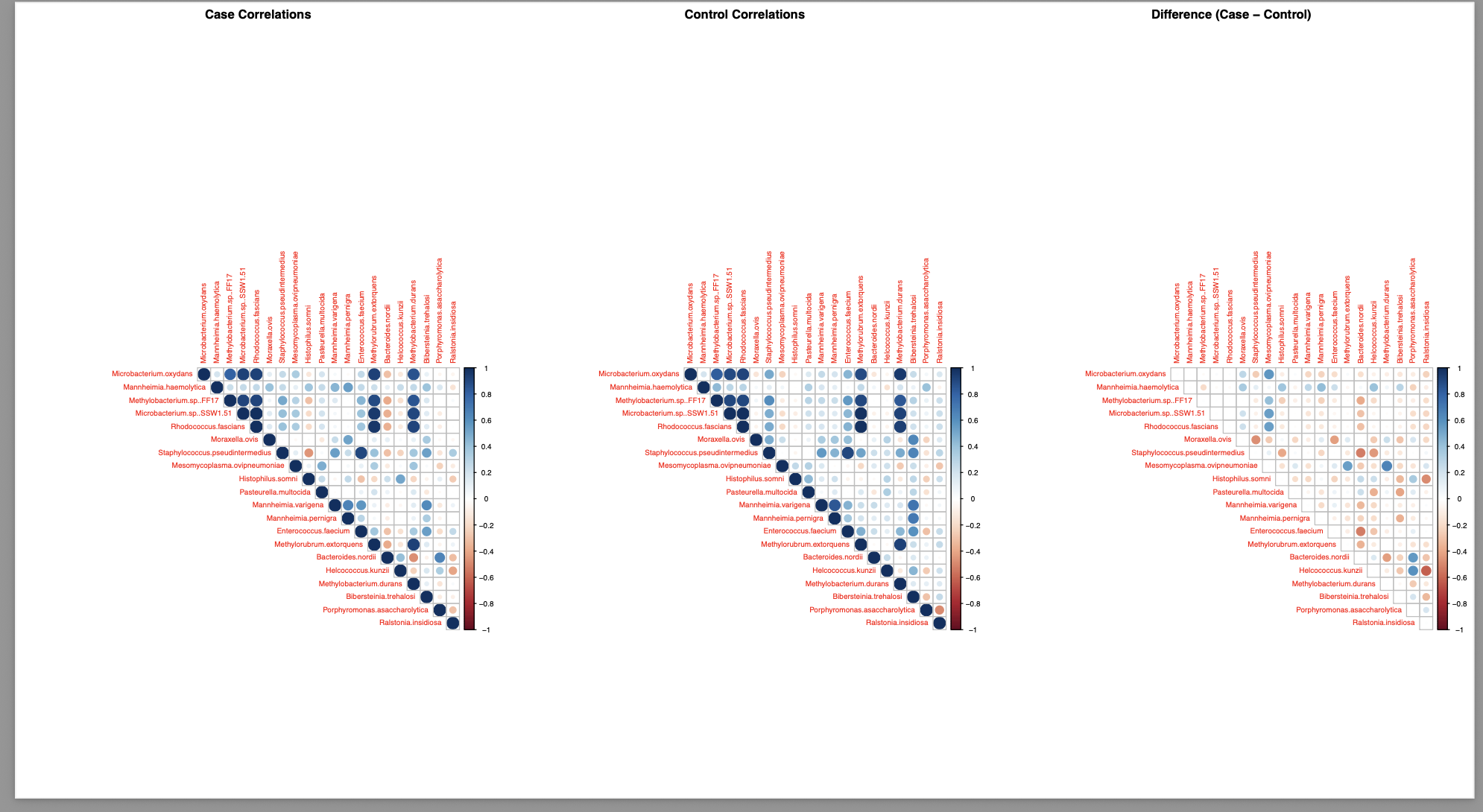


**B**

**A**
